# Non-Synonymous Single Nucleotide Polymorphisms in the P2X Receptor Genes: Association with Diseases, Impact on Receptor Functions and Potential Use as Diagnosis Biomarkers

**DOI:** 10.3390/ijms150813344

**Published:** 2014-07-30

**Authors:** Emily A. Caseley, Stephen P. Muench, Sebastien Roger, Hong-Ju Mao, Stephen A. Baldwin, Lin-Hua Jiang

**Affiliations:** 1 School of Biomedical Sciences, Faculty of Biological Sciences, University of Leeds, Leeds LS2 9JT, UK; E-Mails: bs09e2c@leeds.ac.uk (E.A.C.); s.p.muench@leeds.ac.uk (S.P.M.); s.a.baldwin@leeds.ac.uk (S.A.B.); 2 Inserm U1069, University of Tours, Tours 37032, France; E-Mail: sebastien.roger@univ-tours.fr; 3 State Key Laboratory of Transducer Technology, Shanghai Institute of Microsystem and Information Technology, Chinese Academy of Science, Shanghai 200050, China; E-Mail: hjmao@mail.sim.ac.cn; 4 Department of Physiology and Neurobiology, Xinxiang Medical University, Xinxiang 453003, China

**Keywords:** *P2RX*, NS-SNP, loss- or gain-of-function mutation, disease association, structure-function relationships

## Abstract

P2X receptors are Ca^2+^-permeable cationic channels in the cell membranes, where they play an important role in mediating a diversity of physiological and pathophysiological functions of extracellular ATP. Mammalian cells express seven P2X receptor genes. Single nucleotide polymorphisms (SNPs) are widespread in the *P2RX* genes encoding the human P2X receptors, particularly the human P2X7 receptor. This article will provide an overview of the non-synonymous SNPs (NS-SNPs) that have been associated with or implicated in altering the susceptibility to pathologies or disease conditions, and discuss the consequences of the mutations resulting from such NS-SNPs on the receptor functions. Disease-associated NS-SNPs in the *P2RX* genes have been valuable in understanding the disease etiology and the receptor function, and are promising as biomarkers to be used for the diagnosis and development of stratified therapeutics.

## 1. Introduction

Adenosine triphosphate (ATP) is best known as an intracellular energy supplier for a wide range of activities inside the cell. However, ATP is also released into the extracellular milieu where it acts as a signalling molecule. It was first shown in a seminal study that ATP released from sensory nerves caused vasodilation [1]. The theory of purinergic signalling was posited in 1972 [2] but remained controversial until molecular cloning of the genes encoding the receptors for extracellular ATP in the 1990s [3,4,5,6]. It is now firmly established that two structurally and functionally distinctive families of P2 purinergic receptors in the cell membranes mediate intracellular signalling evoked by extracellular ATP; ligand-gated ion channel P2X receptors and G-protein-coupled P2Y receptors [7].

Mammalian cells express seven genes encoding seven P2X receptor subunits, P2X1–P2X7 [8]. These subunits are capable of forming homomeric receptors on their own, with the exception of the P2X6 subunit, or heteromeric receptors, with P2X1/2, P2X1/4, P2X1/5, P2X2/3, P2X2/5, P2X2/6 and P2X4/6 receptors being defined so far [9]. P2X receptors are expressed in many tissues and cells, where they are important in mediating a diversity of physiological processes from neurotransmission, muscle contraction and hearing to immune responses. P2X receptors function as Ca^2+^-permeable cationic channels that open in response to brief receptor activation. Prolonged activation of many of the P2X receptors, most saliently the P2X7 receptor, can induce formation of large pores that confer permeability of the cell membranes to much larger cations and fluorescent dyes [8]. The cellular distribution, pharmacological properties, and physiological and pathophysiological functions of the P2X receptors have been extensively discussed in several recent reviews [9,10,11,12,13]. The *P2RX* genes encoding the human P2X receptors, particularly the human P2X7 receptor, are highly polymorphic and contain a large set of single nucleotide polymorphisms (SNPs). Genetic association studies support non-synonymous SNPs (NS-SNPs) in the *P2RX* genes as an important genetic factor that alters the susceptibility of individuals to various disease conditions. Characterisation of the mutations in P2X receptors resulting from NS-SNPs have shed light on general and receptor-specific structure-function relationships of these receptors [9]. Furthermore, by combining these approaches, studies of disease-associated NS-SNPs have provided novel insights into disease mechanisms (e.g., [14]). In this short review, we will discuss the NS-SNPs in the *P2RX* genes, focusing on those which are associated with or implicated in various pathologies. We will give a brief introduction of the context and describe the studies that support association of NS-SNPs with particular disease(s). We will also discuss the consequences of such NS-SNP mutations on receptor function in order to facilitate a better understanding of the disease mechanisms and structure-function relationships of P2X receptors. Disease-associated NS-SNPs in the *P2RX* genes are promising as biomarkers to be used for the diagnosis and development of stratified therapeutics.

## 2. Non-Synonymous Single Nucleotide Polymorphisms (NS-SNPs) in the *P2RX* Genes and Disease Association

### 2.1. NS-SNPs in the P2RX2 Gene Increase Susceptibility to Hearing Loss

The human *P2RX2* gene has a chromosomal location of 12q24.33 and is composed of 11 exons [15]. Expression of the P2X2 receptor is found in the cochlea of the inner ear, particularly in spiral ganglion neurons, epithelial cells in Reissner’s membrane, and hair cell stereocilia in the organ of Corti [16,17,18]. Furthermore, there is evidence to suggest that noise up-regulates P2X2 receptor expression in the cochlea [19]. Such observations have led to numerous investigations into the role of the P2X2 receptor in regulating multiple processes in hearing, including auditory neurotransmission [18,20], outer hair cell electromotility [21], K^+^ recycling [22,23], and control of inner ear gap junctions [22]. Overall, these studies have provided clear evidence to support a critical role for the P2X2 receptor in maintaining normal hearing. Consistently, P2X2-deficient mice manifested severe progressive hearing loss and high-frequency hearing loss as a result of exposure to continuous moderate noise in young adulthood [24,25].

Noise-induced hearing loss and age-related hearing loss are the two major forms of hearing loss in humans. DFNA41 is an autosomal dominant and non-syndromic form of deafness which presents as bilateral and symmetrical post-lingual sensorineural hearing loss, often with high frequency tinnitus occurring simultaneously [26]. The age of DFNA41 onset ranges from 12 to 20 years and is progressive throughout the sufferer’s life time, resulting in hearing loss across all frequencies. The gene(s) responsible for the hearing loss have been mapped to a locus between the marker D12S1609 and 12qter [26]. A rare heterozygous allele containing 178G>T in the *P2RX2* gene has recently been discovered through linkage analysis of genomic DNA from DFNA41 members in two unrelated Chinese families, but not in a large group of control subjects [25]. Moreover, this NS-SNP, resulting in the substitution of valine with leucine at position 60 (V60L) in the human P2X2 receptor, confers loss of receptor function [25]. The members of these two families who were heterozygous for the 178G>T or carried the V60L mutation exhibited significantly greater high-frequency hearing loss following noise exposure in young adulthood [25]. Another recent linkage analysis study of an Italian family has identified a distinctive NS-SNP, 1057G>C [27]. This NS-SNP changes glycine at position 353 to arginine (G353R) in the human P2X2 receptor.

### 2.2. NS-SNP in the P2RX4 Gene

#### 2.2.1. 1248A&gt;G Is Associated with High Pulse Pressure

The human *P2RX4* gene is located on chromosome 12q24.31 [28,29] and has 12 exons. Expression of the P2X4 receptor has been documented in endothelial cells. Activation of the P2X4 receptor induced by ATP, released from vascular endothelial cells in response to fluid shear stress, can lead to extracellular Ca^2+^ influx. This subsequently induces the generation of nitric oxide and vessel dilation [30,31,32]. Genetic deletion of the P2X4 receptor in mice impaired blood flow-dependent control of vascular tone and loss of adaptive vascular remodelling (*i.e.*, decrease in vessel size in response to a chronic decrease in blood flow) [33]. These studies have underscored a critical role for the P2X4 receptor in the mechanisms that enable vascular remodelling in response to changes in blood flow.

Pulse pressure, the difference between systolic and diastolic blood pressures, is dependent on the elastic properties of large arteries as well as cardiac stroke volume, and has gained increasing recognition as an important and independent indicator of morbidity and mortality in cardiovascular diseases. A recent study has genotyped two Australian cohorts composed of 430 and 2874 subjects respectively and identified the 1248A>G polymorphism (rs28360472) as a potential risk factor for high pulse pressure [34]. This NS-SNP results in the substitution of tyrosine at position 315 with cysteine in the human P2X4 receptor, which impairs receptor function. Analysis of the second cohort suggests an association between the 1248A>G polymorphism and high pulse pressure [34].

#### 2.2.2. 1248A&gt;G Increases Susceptibility to Age-Related Macular Degeneration (AMD)

Macular degeneration is a major cause of blindness amongst the elderly [35]. The pathogenesis of age-related macular degeneration (AMD) includes genetic changes which compromise the innate immunity required for clearance of debris in the retina. The P2X4 receptor, together with the P2X7 receptor, which will be introduced in the next section, is found in microglia and macrophages in the central nervous system (CNS) and in the eye [36,37]. A recent study has investigated the 1248A>G polymorphism in the *P2RX4* gene and eleven NS-SNPs in the *P2RX7* gene in a cohort of 744 Caucasian AMD patients and 557 control subjects [36]. This study found an association of the 1248A>G polymorphism in the *P2RX4* gene, but none of the NS-SNPs in the *P2RX7* gene, with increased susceptibility to AMD. The P2X7 receptor is known to exhibit significant scavenger receptor activity [38]. P2X7 receptor-mediated phagocytosis was strongly suppressed in cells co-expressing the P2X7 receptor with the P2X4 receptor carrying the Y315C mutation [36], supporting the notion that a functional partnership between these two receptors is important in maintaining the scavenger function of macrophages and microglia in the eye.

### 2.3. NS-SNPs in the P2RX7 Gene

The human *P2RX7* gene shares approximately the same chromosomal location as the *P2RX4* gene, within 130 kb based on radiation hybrid mapping, and contains 13 exons [28]. The P2X7 receptor is widely expressed in immune cells such as monocytes, macrophages, mast cells, lymphocytes, leukocytes, and dendritic cells. It is important in underpinning the role of extracellular ATP in immune responses, including the production of pro-inflammatory cytokines, such as interleukin (IL)-1β, and elimination of dead cells and intracellular pathogens, and also in stimulating cell proliferation and cell death [39]. The P2X7 receptor is also expressed by microglia, astrocytes and oligodendrocytes in the CNS and plays a role in glia-neuron interactions via mediating the release of neurotransmitters as well as the generation of IL-1β from microglia [11,39]. The P2X7 receptor present on satellite glial cells of the dorsal root ganglions (DRG) in the peripheral nervous system (PNS) has a similar role in mediating ATP release. Furthermore, the P2X7 receptor is found on bone cells. In osteoblasts the receptor is engaged in the activation of downstream extracellular signal-regulated kinase as a result of fluid flow and stimulation of mineralisation [40,41,42,43]. In osteoclasts the P2X7 receptor has been implicated in the formation of multinucleated cells, apoptosis, NF-κB activation, and it is required for intercellular Ca^2+^ signalling between osteoclasts and osteoblasts [44]. In P2X7-KO mice, the trabecular bone resorption in the tibias was increased, but the overall and cortical bone content, periosteal circumference in the femurs, and periosteal bone formation were reduced [45] and the effect of mechanical loading on periosteal bone formation was also attenuated [42]. These findings support a crucial role for the P2X7 receptor in bone formation, metabolism and remodelling.

A number of studies of SNPs in the *P2RX7* gene have been carried out over a period of more than a decade in order to examine a potential role of the human P2X7 receptor in the aetiology of a variety of clinical conditions, including chronic lymphocytic leukaemia (CCL) [46,47,48,49,50,51], affective mood disorders [52,53,54,55], multiple sclerosis [56], chronic pain [14], systemic lupus erythematosus, rheumatoid arthritis [57,58,59], childhood febrile seizure [60], ischemic stroke, ischemic heart disease [61], tuberculosis [62,63,64,65,66], sepsis [67], toxoplasmosis [68,69], osteoporosis, and bone fracture [70,71,72,73,74]. Table 1 provides a brief summary of the NS-SNPs that have been associated with or implicated in altering susceptibility to particular disease condition(s). One point of note is that the 1513A>C polymorphism was proposed in earlier studies to be associated with CLL [46,47], but such association has not been supported by subsequent studies [48,49,50,51]. In several other cases the supporting evidence remains limited. In the following section we will discuss the studies that support association ofNS-SNPs in the *P2RX7* gene with disease(s).

#### 2.3.1. 1405A&gt;G and 1068G&gt;A Are Implicated in Susceptibility to Affective Mood Disorders

Affective mood disorders, including bipolar disorder (BD), major depressive disorder (MDD) and anxiety disorder (AD), define a large group of common depression conditions, all characterized by consistent and pervasive alterations in mood that change thoughts, emotions and behaviours and thereby result in social impairments [75,76,77,78,79]. The *P2RX7* gene has been identified as a locus of susceptibility to affective mood disorders [80]. Several groups have conducted genetic association analysis of SNPs in the *P2RX7* gene in order to probe the role of the human P2X7 receptor in the pathogenesis of affective mood disorders. The first two studies were reported in 2006, one examining a cohort consisting of 231 Canadian BD patients (182 type-I and 31 type-II) and 214 control subjects [52], and the other study a cohort comprising 1000 German Caucasian patients diagnosed with MDD and 1029 control subjects [53]. Both studies have revealed an association of the 1405A>G polymorphism for the Q460R mutation with BD [52] and MDD [53]. A subsequent study of 604 BD patients and 560 control subjects with UK and Irish background has confirmed this association [55]. This study, when combined with the previous studies by Barden *et al.* [52] and Lucae *et al.* [53], has found an even more robust association with affective mood disorders. Another study analysing 171 Hungarian patients with MDD, type-I and type-II BD and 178 control subjects also supports the association of the 1405A>G polymorphism with affective mood disorders [81]. However, no significant association with the 1405A>G polymorphism was observed in a UK cohort of 687 patients with type-I BD, 1036 patients with unipolar recurrent major depression and 1204 control subjects [82] and also in a cohort of 1445 type-I BD patients and 2006 control subjects from four Central and Eastern European countries (Germany, Poland, Romania, and Russia) [83]. A meta-analysis study of 6962 patients and 9262 control subjects from 13 different studies deposited in various databases has also concluded no association of the 1405A>G polymorphism with affective mood disorders overall, but has found significant association in family-based cohorts [84]. A study examining a cohort of 179 Caucasian patients with AD and syndromal panic attacks and 462 control subjects has shown a trend for association of the 1068G>A polymorphism, encoding the gain-of-function A348T mutation, with AD [54]. However, several recent large-scale genome-wide association studies have failed to identify *P2RX7* as a locus of susceptibility to BD [85,86,87]. Future studies of large and clearly annotated samples are required to clarify or validate the association of these NS-SNPs in the *P2RX7* gene with affective mood disorders.

#### 2.3.2. 370T&gt;C and 489C&gt;T Increase Susceptibility to Multiple Sclerosis (MS)

Multiple sclerosis (MS) is a CNS neurodegenerative disease, characterized by white matter lesions with demyelination, inflammation and axonal degeneration [88]. Increased P2X7 expression has been found in lesion sites in the spinal cord and brain of MS patients [89,90]. A study, using experimental autoimmune encephalomyelitis of mice, a rodent model of human MS, has shown that inhibition of the P2X7 receptor using selective antagonists can reduce demyelination as well as improving MS-associated neurological deficits, thus providing strong evidence to support a role for the P2X7 receptor in the pathogenesis of MS [91]. A recent study has examined a cohort of 734 MS Spanish patients and found that the 370T>C and 489C>T polymorphisms are prevalent in these patients and associated with an increased susceptibility to MS [56]. These two NS-SNPs introduce gain-of-function A76V and H155Y mutations in the human P2X7 receptor.

#### 2.3.3. 489C&gt;T and 835G&gt;A Alter Chronic Pain Sensitivity

It has long been known that extracellular ATP is nociceptive. The P2X2/3 receptor, which is exclusively expressed in DRG neurons, mediates ATP-induced pain and inflammatory and neuropathic pain [13]. The P2X4 receptor also contributes to chronic neuropathic and inflammatory pain with its up-regulated expression in the activated spinal microglia following peripheral nerve injury being critical in the development of neuropathic pain [13]. Moreover, the P2X4 receptor in tissue-residing macrophages is critically involved for inflammatory pain [13]. As mentioned above, the P2X7 receptor mediates ATP-induced IL-1β release from immune cells and glia-neuron interactions and therefore plays an important role in chronic inflammatory and neuropathic pain. Consistently, studies using rodent models have shown that P2X7 deficiency or pharmacological inhibition of the P2X7 receptor can attenuate neuropathic and inflammatory pain [92,93]. It is worth mentioning that the P2X7 receptor expressed in DRG satellite glial cells plays a role in attenuating pain. This is achieved by constituting an inhibitory mechanism along with the P2Y1 receptor in DRG neurons in order to down-regulate P2X3 protein surface expression on DRG neurons, thereby reducing the pain signalling mediated by P2X receptors containing the P2X3 subunit [94].

A recent genome-wide study of SNPs in the genes encoding the mouse and human P2X7 receptors has provided further support for a critical role of the P2X7 receptor in chronic pain [14]. More importantly, detailed analysis of the effect of a pain-related NS-SNP on the functional properties of the P2X7 receptor has shed light on the disease mechanism at the receptor level. This study first assessed a number of SNPs in several genes in relation to the severity of nerve injury-induced tactile hypersensitivity or mechanical allodynia in 18 strains of mice. One of the NS-SNPs identified in the mouse *p2rx7* gene, which was strongly associated with altered sensitivity to chronic neuropathic pain, introduces P451L mutation in the *C*-terminal tail of the mouse P2X7 receptor. Mice expressing the Leu^451^-containing P2X7 receptor were substantially less sensitive to neuropathic pain than mice expressing the Pro^451^-containing P2X7 receptor. Further functional characterizations of the P2X7 receptors expressed in macrophage cells from these mice indicate that the P451L mutation strongly impairs the large pore-forming functionality, without altering ion channel function. These results suggest that the ability of the P2X7 receptor to induce or form large pores has a critical role in determining the sensitivity to chronic neuropathic pain. The same study also examined several SNPs in the human *P2RX7* gene in two different cohorts of patients with chronic inflammatory pain [14]. In the first cohort composed of over 300 women with breast surgery, approximately half developed chronic pain following the procedure; the 489C>T polymorphism resulting in the gain-of-function H155Y mutation was strongly correlated with higher levels of post-mastectomy pain and, by contrast, the 835G>A polymorphism for the loss-of-function R270H mutation was associated with lower levels of pain. A significant association of the 835G>A polymorphism with chronic pain was also identified in the second cohort of patients with osteoarthritis, an inflammatory condition.

#### 2.3.4. 489C&gt;T Is a Risk Factor for Childhood Febrile Seizure

The important role of the P2X7 receptor in inflammatory responses has led to a recent investigation of SNPs in the human *P2RX7* gene in association with childhood febrile seizures [60]. This study has examined 157 febrile seizure patients and 163 control subjects and identified the 489C>T polymorphism, which leads to the H155Y mutation, as a risk factor for febrile seizures. Such genetic association was further verified by screening febrile seizure patients from the Wellcome Trust Case Control Consortium 1958 Birth Cohort.

#### 2.3.5. 1513A&gt;C Reduces Cardiovascular Risk

Similarly, the implication of P2X7 receptor-mediated inflammation in atherothrombosis has prompted the study of SNPs in the human *P2RX7* gene as a cardiovascular risk factor in patients with ischemic heart disease and ischemic stroke [61]. The study genotyped SNPs in the *P2RX7* gene in 1244 patients with ischemic heart disease and 5969 individuals with cardiovascular risk factors as well as 2488 control subjects, and detailed analysis supports that the 1513A>C polymorphism or the loss-of-function E496A mutation may reduce the risk of ischemic heart disease in smokers. Such an association was however not obtained in non-smokers. In addition, this study analysed 4138 ischemic stroke patients and 2528 control subjects, and found that this same NS-SNP may also reduce the risk of ischemic stroke.

#### 2.3.6. 1513A&gt;C in Susceptibility to Tuberculosis (TB)

Tuberculosis (TB) is a disease that arises from infection by the intracellular pathogen *Mycobacterium tuberculosis* (MTB). MTB predominantly replicates in macrophage cells, which act as the main host cells regulating MTB growth and viability. Macrophage cells can kill MTB by producing reactive oxygen or nitrogen species [95], and it is known that the P2X7 receptor plays a role in the ATP-induced macrophage killing of MTB [96,97,98]. It is this role in MTB killing that has driven several genetic association studies to investigate the connection between SNPs in the *P2RX7* gene and susceptibility to TB in various populations. These studies so far have reported inconsistent findings. For example, an association of the 1513A>C polymorphism for the E496A mutation with TB was not found in an earlier study examining more than 300 Gambian TB patients and 160 control subjects [62]. However, the 1513A>C polymorphism has been identified as a risk factor in a separate study that analyzed two relatively small cohorts of individuals from Southeast Asia including Vietnam, Cambodia, and Laos (86 TB patients and 167 control subjects in the first cohort, and 99 TB patients and 102 control subjects in the second cohort) [63]. This NS-SNP has also been found to increase the susceptibility to TB in Mexican populations [64], but not in Chinese and Asian Indian subjects [65,66]. A meta-analysis study attempted to address the inconsistency of the results obtained from these studies, and has concluded significant association of the 1513A>C polymorphism with an increased susceptibility to TB [99]. Further analysis of subgroups indicates that this NS-SNP is associated with a higher susceptibility to TB in Asian populations but is not a risk factor in Latino or African populations [99]. There are a number of factors that may compound these findings, including the allele frequency in different ethnic groups, variations in the age of the subjects examined, and TB being pulmonary or extrapulmonary.

#### 2.3.7. 489C&gt;T and 1405A&gt;G Are Potential Factors Increasing Susceptibility to Sepsis

Sepsis is caused by an overactive inflammatory response to infection. One recent study employing the multi-individual array platform, capable of simultaneously identifying variations in the same nucleotide in thousands of samples, screened DNA from blood samples taken from 95 patients with severe sepsis and 518 control subjects [67]. This study found that the prevalence of two NS-SNPs, 489C>T and 1405A>G for the H155Y and Q460R mutations respectively, were significantly higher in patients suffering from severe sepsis following surgical trauma, implying the presence of these NS-SNPs as a potential risk factor for sepsis. Further studies are required to ascertain the association.

#### 2.3.8. 1068G&gt;A and 1513A&gt;C Alter Susceptibility to Toxoplasmosis

*Toxoplasma gondii* is an obligate intracellular parasite, affecting over one third of the human population. Despite most infections being asymptomatic and self-limiting, infection acquired during pregnancy can lead to congenital toxoplasmosis, resulting in neonatal death or fetal abnormalities. Affected infants may display defects in brain and eye functions, and the damage induced by this parasite appears to occur at a point when the foetal immunity is poorly developed [100,101]. A study, examining SNPs in 149 groups consisting of the child and their parents, has identified the 1068A allele, or the 1068G>A polymorphism for the gain-of-function A348T mutation, as a genetic factor reducing the susceptibility to the conditions such as intracranial calcification, hydrocephalus and retinochoroiditis resulting from *Toxoplasma gondii* infection *in utero* [68]. Consistently, a separate study has shown that macrophages expressing the loss-of-function E496A mutant P2X7 receptor, due to the 1513A>C polymorphism, were less capable of ATP-induced killing of *Toxoplasma gondii* [69]. These findings provide consistent evidence to support an inverse relationship between the P2X7 receptor activity and the susceptibility to toxoplasmosis.

#### 2.3.9. Multiple NS-SNPs Are Associated with Osteoporosis and Bone Fracture Risk

Osteoporosis is a progressive skeletal disease causing low bone mass or bone mineral density (BMD) and microarchitectural deterioration of bone tissues, predisposing individuals with increased bone fragility and fracture risk [102,103]. As introduced above, the P2X7 receptor has a critical role in bone formation, metabolism and remodelling, leading to several genetic association studies of SNPs in the human *P2RX7* gene with increased susceptibility to osteoporosis or risk of bone fracture. These studies have identified an association with several NS-SNPs (Table 1). The first study examined a cohort of 1764 postmenopausal women and has reported that two NS-SNPs, 1513A>C and 1729T>A, which cause the loss-of-function E496A and I568N mutations respectively, were associated with the vertebral fracture incidence rate over 10 years [70]. The second study investigated a cohort of 506 post-menopausal women from the Aberdeen Prospective Osteoporosis Screening Study in relation to BMD in the lumbar spine [71]. Patients carrying the 946G>A polymorphism for the loss-of-function R307Q mutation showed significantly lower lumbar spine BMD, both at baseline and 6–7 years later. In addition, a group of subjects carrying multiple NS-SNPs (946G>A, 1096C>G, 1513A>C and 1929T>A, resulting in the loss-of-function R307Q, T357S, E496A, and I568N mutations respectively) manifested approximately 9% reduction in their lumbar spine BMD each year over a period of 10 years. Another study was published at the same year, which analyzed 12 NS-SNPs with respect to the risk of bone fracture in a cohort of 1694 women participating in the Danish Osteoporosis Prevention Study [72]. This study, combining measurements of both BMD and fracture incidence at baseline and after 10 years, has also found the 946G>A polymorphism to exhibit a clear association with an increased rate of bone loss, with the rate of bone loss being 40% higher in patients heterozygous for this polymorphism. Furthermore, the 1729T>A polymorphism for the I568N mutation has also been associated with an increased rate of bone loss. In contrast, another two NS-SNPs, 1068G>A and 1405A>G, giving rise to the A348T and Q460R mutations, were linked with an increased BMD and a lower rate of vertebral fracture incidence 10 years after menopause. Two further studies have examined a Dutch cohort of 690 women and 231 men, and a Danish cohort of 462 osteoporotic men and women. In the Dutch cohort, the 1068G>A polymorphism for the gain-of-function A348T mutation was found to be associated with higher BMD at the lumbar spine. In contrast, the 474G>A and 1513A>C polymorphisms for the loss-of-function G150R and E496A mutations respectively were correlated with decreased BMD in the hip, and the 1405A>G polymorphism for the Q460R mutation was linked to a strong increase in the risk of developing osteoporosis [74]. The second recent study examined NS-SNPs as a risk factor of bone fracture [73]. The 474G>A polymorphism for the loss-of-function G150R mutation is associated with a lower hip BMD in both men and women, whereas the 1513A>C polymorphism for the loss-of-function E496A mutation was linked with lower lumbar spine BMD in women and lower hip BMD in men. In contrast, the alleles encoding the gain-of-function mutations were correlated with lower fracture risk and/or increased BMD. The 1405A>G polymorphism for the Q460R mutation was associated with increased BMD in the hips of women, and the 1068G>A polymorphism for the A348T mutation was linked to increased BMD and lower fracture in men. This is the first study to show segregation of NS-SNPs between the genders and suggest that the effects on BMD and bone fracture are mainly driven by the 1068G>A polymorphism in men and by the 1405A>G and 1513A>C polymorphisms in women [73]. Overall, these genetic studies of NS-SNPs support the concept that increased P2X7 receptor activity confers a lower rate of bone loss and a reduced risk of bone fracture in both men and women. Consistent with these genetic association studies in humans, the NS-SNP in the mouse *p2rx7* gene for the loss-of-function P451L mutation has been found to be associated with an increased predisposition to osteoporosis [104].

**Table 1:** Disease-associated non-synonymoussingle nucleotide polymorphisms (NS-SNPs) in the *P2RX7* gene.

rs Number	Change in Nucleotide (Amino Acid) Sequence	Implicated Conditions
rs17525809	370T>V (A76V)	Multiple sclerosis [56]
rs28360447	474G>A (G150R)	Osteoporosis [73,74]
rs208294	489C>T (H155Y)	Multiple sclerosis [56]; chronic pain [14]; severe sepsis [67]; children febrile seizures [60]
rs7958311	835G>A (R270H)	Chronic pain [14]
rs28360457	946G>A (R307Q)	Osteoporosis [71,72]
rs1718119	1068G>A (A348T)	Osteoporosis [72,73,74]; anxiety disorder [54]; toxoplasmosis [68]
rs2230911	1096C>G (T357S)	Osteoporosis [71]
rs2230912	1405A>G (Q460R)	Osteoporosis [72,73,74]; severe sepsis [67]; bipolar disorders and major depressive disorders [52,53,55,81] (but see [82,83]) ^†^
rs3751143	1513A>C (E496A)	Osteoporosis [70,71,72,73,74]; tuberculosis [63,64,99]; cardiovascular risks [61]
rs1653624	1729T>A (I568N)	Osteoporosis [70,71,72]

^†^, association with bipolar disorders and major depressive disorders is not supported by studies shown in brackets.

## 3. NS-SNP Mutational Effects on Receptor Function

**Figure 1:**
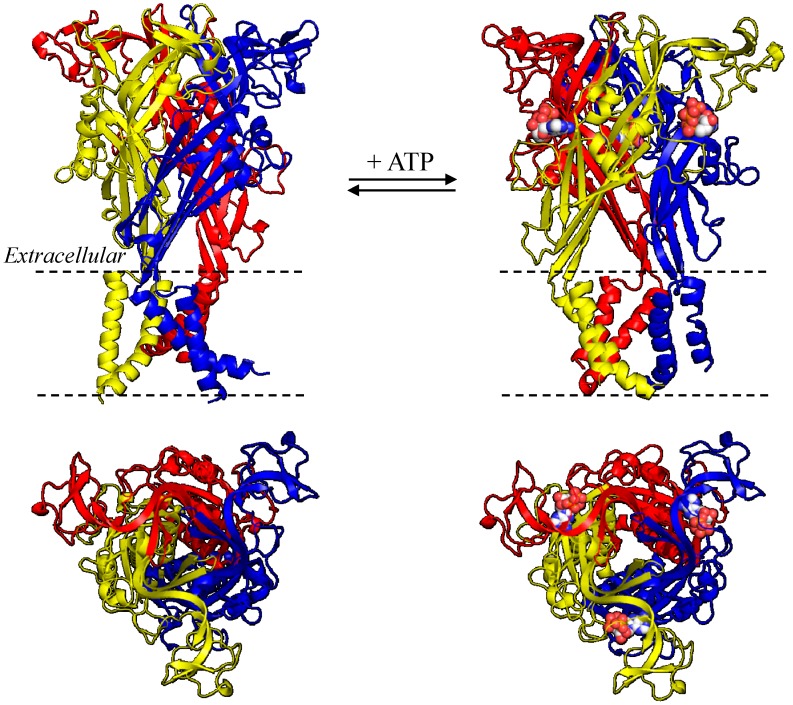
Structural homology models of the trimeric human P2X7 receptor. (**Left**) closed state; and (**right**) ATP-bound open state, viewed parallel to the plasma membrane (**top**) or the extracellular side (**bottom**). The three subunits are denoted in different colours, and the three bound ATP molecules are highlighted in spheres.

**Figure 2:**
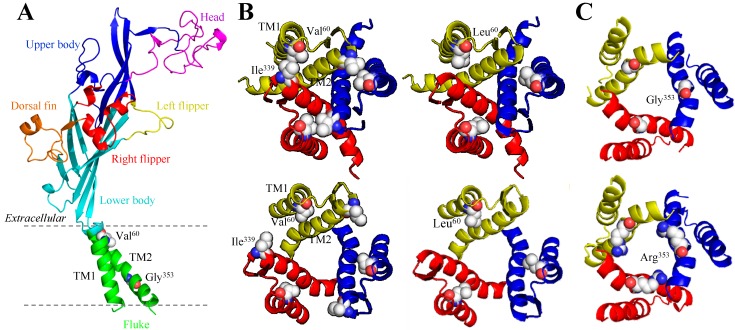
Structural homology models of the human P2X2 receptor. (**A**) The dolphin-like structure of a single receptor subunit in the closed state is shown with distinctive body parts in different colours, and Val^6^^0^ in the extracellular end of TM1 and Gly^353^ in the intracellular half of TM2 are highlighted; (**B**) Changes in the distance between Val^6^^0^ and Ile^339^ (**left**) and structural differences introduced by the V60L in the closed (**top**) and open states (**bottom**) viewed from the extracellular side of the membrane; and (**C**) Structural differences introduced by mutating Gly^353^ (**top**) to arginine (**bottom**) in the open state viewed from the cytoplasmic side of the membrane. TM: transmembrane domains.

**Figure 3:**
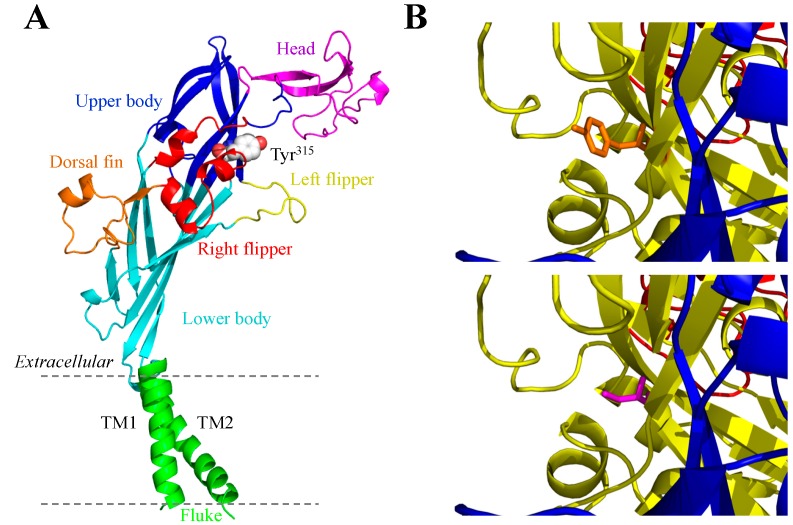
Structural homology models of the human P2X4 receptor. (**A**) the dolphin-shaped structure of a single receptor subunit in the closed state shown with distinctive body parts in different colours (the same colour scheme used in Figure 2A for the human P2X2 receptor subunit) and the location of Tyr^315^ highlighted;and (**B**) views of Tyr^315^ (orange; **top**) and Cys^315^ (magenta, **bottom**) in the close proximity to the ATP-binding site in the open state.

**Figure 4:**
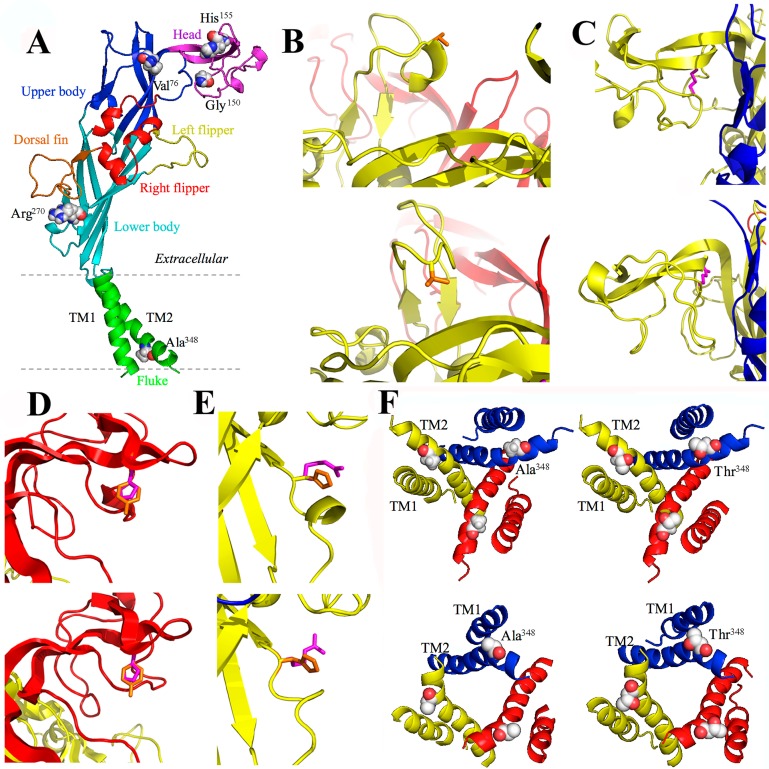
Structural homology models of the human P2X7 receptor. (**A**) the dolphin-shaped structure of a single receptor subunit in the closed state is shown with distinctive body parts in different colours (the same colour scheme used in Figure 2A and Figure 3A for the human P2X2 and P2X4 receptor subunits) and location of the residues to be mutated by disease-associated NS-SNPs are highlighted; (**B**–**F**) structural differences introduced by the V76A (**B**), G150R (**C**), H155Y (**D**), R270H (**E**), and A348T mutation (**F**) in the closed (**top**) and open state (**bottom**). The original residues are shown in orange and the substituted residues in magenta in (**B**–**E**). (**F**) is viewed from the cytoplasmic side of the membrane.

The seven mammalian P2X receptor subunits vary in length, with the human P2X receptor subunits ranging from 377 amino acid residues in the P2X6 receptor to 595 residues in the P2X7 receptor [8]. However, all of them have the same membrane topology, containing a large extracellular domain, two transmembrane domains (TM1 and TM2) and intracellular *C*- and *N*-termini (Figure 1, Figure 2, Figure 3 and Figure 4). While the extracellular and transmembrane domains show strong amino acid sequence conservation and are similar in length, the intracellular domains are less conserved and variable in length, with the P2X7 receptor subunit having a much longer *C*-terminus. The crystal structures of the zebrafish P2X4 receptor have been determined in the closed [105] and ATP-bound open states [106], representing the most recent milestone in P2X receptor research. Despite the lack of the intracellular domains, these structures have been invaluable in understanding ATP binding and channel gating [107]. The high sequence similarity between the zebrafish P2X4 receptor and the mammalian P2X receptors enables homology modelling of the latter receptors. Such structural models have provided the structural framework for more accurate interpretations of the site-directed mutagenesis data accumulated over the past decade [107,108,109] and provide exciting opportunities to define the structure-function relationships of the mammalian P2X receptors in greater detail [107] and design small molecular chemicals for therapeutics. As illustrated in Figure 1, the human P2X7 receptor, as with other P2X receptors, assumes a “chalice-like” structure, in which three subunits are intertwined around each other in a three-fold symmetric manner running perpendicularly to the cell membrane and the six α-helical transmembrane domains, two from each of the three subunits, form the central aqueous ion-conducting pore. The overall shape of each subunit has been suggested to resemble that of a leaping dolphin (Figure 2A–Figure 4A).

The mutations resulting from NS-SNPs have been examined in terms of their effects on the Ca^2+^-permeable cationic channel function by measuring agonist-induced Ca^2+^ influx or currents and on the ability to induce large pore formation by monitoring fluorescent dye uptake. In this section we will discuss such mutational effects, focusing on those arising from the disease-associated NS-SNPs described above. Their locations in the structural homology models are shown for the human P2X2 receptor (Figure 2A), human P2X4 receptor (Figure 3A), and human P2X7 receptor except for the mutations (Glu^46^^0^, Glu^496^ and Ile^568^) in the *C*-terminus of the human P2X7 receptor (Figure 4A) as the structural information of the intracellular domains is still unavailable.

### 3.1. V60L and the Human P2X2 Receptor

The effect of the V60L mutation has been examined in several heterologous expression systems. When expressed in HEK293 cells, the human V60L mutant P2X2 receptor failed to mediate detectable ATP-induced currents, in contrast with the strong currents conducted by the wild-type receptor [25]. Expression of the wild-type P2X2 receptor in MDCK-II cells conferred strong ATP-induced FM1-43 fluorescent dye uptake but expression of the mutant V60L receptor resulted in no such dye uptake. Cells co-expressing the wild-type and mutant P2X2 subunits exhibited ATP-evoked FM1-43 uptake but the response was reduced by approximately 60% [25]. The GFP-tagged wild-type or V60L mutant P2X2 proteins, when expressed in the explant cultures of neonatal rat organ of Corti and vestibular tissues, showed identical localization and, as anticipated, appeared in the apical membranes of hair cells in the organ of Corti [25]. These results suggest that the V60L mutation impairs the function of the receptor rather than its expression or subcellular localization. Valine at this position is highly conserved within the mammalian P2X2 receptors and located at the outer end of TM1 (Figure 2B). Cysteine substitution of the corresponding residue (Val^48^) in the rat P2X2 receptor had minimal effect on receptor function [110]. Simultaneous cysteine substitution of Val^48^ and Ile^328^ in the outer end of TM2 resulted in the spontaneous formation of a disulphide bond which prevented the channel opening, suggesting close vicinity of these two positions and substantial relative movement of the regions surrounding them during receptor activation [110]. This notion has been elegantly illustrated in structural models, which show the two positions to juxtapose very closely in the closed state but move far apart in the open state (Figure 2B). The structural models offer one possible interpretation of the loss of function by the V60L mutation; the larger side-chain of leucine introduced at this position may impede the relative movement of TM1 and TM2 (Figure 2B), the conformational changes required for channel opening.

### 3.2. G353R and the Human P2X2 Receptor

Gly^353^ is located in the intracellular half of TM2 (Figure 2A,C). This residue is completely conserved in the mammalian P2X receptor family, corresponding to Gly^342^ in the rat P2X2 receptor [111]. A previous study which combined sited-directed mutagenesis and structural modelling suggests that in the rat P2X2 receptor the residues between Asn^333^ and Asp^349^ form the narrowest part or the physical gate of the ion-permeating pore that occludes the ion permeation in the closed state and Gly^342^ is thus part of the intracellular pore [111]. The mutational effect on the human P2X receptor function has not been characterized so far. It has been proposed based on structural modelling that the mutation of Gly^353^ to arginine with a positively-charged and long side-chain may introduce structural distortions and anomalous interactions with the membrane lipids [27], but the supporting evidence still awaits. Nonetheless, Gly^342^ in the rat P2X2 receptor has been examined in considerable detail. Expression of the G342C mutant receptor resulted in small spontaneous currents, as well as ATP-evoked currents [111,112]. The P2X2 receptor carrying substitution with alanine, aspartate, serine, threonine, tryptophan, or phenylalanine exhibited normal channel unitary conductance, which was substantially reduced by the G342K mutation [111]. Introduction of the G353R mutation in the human P2X2 receptor may confer a larger inhibitory effect on the channel conductance.

### 3.3. Y315C and the Human P2X4 Receptor

The effects of the Y315C mutation on the human P2X4 receptor have been characterized after heterologous expression in HEK293 cells using patch-clamp recording [34]. The Y315C mutation impaired receptor function by conferring 10-fold reduction in the maximal current amplitude and >30-fold reduction in the ATP sensitivity. Replacement of Tyr^315^ with serine carrying an OH group instead of a SH group in cysteine resulted in a similar reduction in the ATP sensitivity with no significant change in the maximal current amplitude. Treatment with the reducing agent dithiothreitol had no effect on ATP-induced currents mediated by the wild-type or Y315S mutant receptor, but increased the currents by the Y315C mutant receptor with the maximal current amplitude similar to that at the wild-type and Y315S mutant receptors. These results suggest that cysteine introduced by the Y315C mutation forms aberrant disulphide bonds with cysteine residues present in the extracellular domain and that such disulphide bonds alter the tertiary structure of the P2X4 receptor and thereby prevent full receptor activation. Structural models of the human P2X4 receptor based on the crystal structure of the zebrafish P2X4 receptor in the closed and open states [105,106] show that position 315 is in close vicinity to the inter-subunit ATP-binding site (Figure 3). Furthermore, molecular docking studies suggest that the replacement of tyrosine with cysteine or serine impairs ATP binding [34].

### 3.4. A76V and the Human P2X7 Receptor

The literature contains a number of studies comparing the effects of either the A76V or V76A mutation with their respective parental “wild-type” human P2X7 receptor carrying alanine or valine at position 76 [56,113,114]. HEK293 cells expressing the A76V mutant receptor exhibited higher Ca^2+^ influx, larger whole-cell currents and an increased rate of ethidium dye uptake when compared to cells expressing the wild-type P2X7 receptor carrying Ala^76^ upon stimulation with ATP [56]. Conversely, ATP-evoked currents and ethidium dye uptake were significantly reduced in HEK293 cells expressing the V76A mutant receptor compared to cells expressing the wild-type receptor harbouring Val^76^, with no major change in the ATP sensitivity [113,114]. Therefore, these studies have drawn a consistent conclusion that the receptors having valine at position 76 show greater functional activity than the receptors carrying alanine. Position 76 is located within the “upper body” region of the extracellular domain in the structural models (Figure 4B) and in the middle of a loop that differs between the P2X7 receptor and the other P2X receptors, including the P2X2 and P2X4 receptors (*c.f.*, Figure 2A–Figure 4A). The change from alanine to valine increases the size of the side-chain (Figure 4B). Comparison of the structures of the zebrafish P2X4 receptor in the closed and open states indicates a lack of substantial conformational change in the upper body during receptor activation [106] and thus the homology modelling of the human P2X7 receptor does not provide obvious clues as to how the A76V mutation increases receptor function.

### 3.5. G150R and the Human P2X7 Receptor

Gly^15^^0^ is highly conserved across all members of the P2X family [8], suggesting a vital role for this residue. Monocytes endogenously expressing the G150R mutant human P2X7 receptor exhibited strongly reduced dye uptake or pore formation as compared to cells expressing the wild-type receptor [115]. Heterologous expression of this mutant P2X7 receptor in HEK293 cells resulted in no detectable current and dye uptake in response to ATP [115]. This residue is situated on the top of the head domain, towards the ‘beak’ part, which undergoes little movement during ATP-induced transition from the closed to open state (Figure 4C). Homology modelling of the human P2X7 receptor thus offers no straightforward interpretation for the loss of receptor function. Nonetheless, arginine has a large and positively-charged side-chain and the G150R mutation may prevent the strucutural flexibility that is required for conformational changes in the adjacent parts [116] (Figure 4C).

### 3.6. H155Y and the Human P2X7 Receptor

While the 489C>T polymorphism for the H155Y mutation is associated with chronic pain, but not with CLL, systemic lupus erythematosus and rheumatoid arthritis, lymphocytes from patients with such conditions expressing the H155Y mutant receptor showed higher Ca^2+^ influx in response to extracellular ATP [58,117]. Such gain of function was consistently observed in heterologous expression cells expressing the mutant receptor. There were significantly greater ATP-induced Ca^2+^ influx and ethidium uptake in HEK293 cells expressing the H155Y mutant receptor [118]. The H155Y mutation also increased agonist-induced currents mediated by the human P2X7 receptor expressed in HEK293 cells [113,119]. The increases in receptor-mediated responses were not related to an effect on the ATP sensitivity, consistent with the location of His^155^ in the head domain far away from the ATP-binding site (Figure 4D). A more systematic study of the effects of changing His^155^ to residues with different side-chain properties on the P2X7 receptor function and cell surface expression has led to the conclusion that the gain of function accompanying the H155Y mutation predominantly results from a higher level of receptor expression on the cell surface [119]. A recent study suggests that the mutation may increase total protein expression of the P2X7 receptor [120].

### 3.7. R270H and the Human P2X7 Receptor

The human P2X7 clone that is widely used in heterologous expression studies encodes a receptor containing histidine instead of arginine at position 270. HEK293 cells expressing the H270R mutant receptor showed larger ATP-induced ethidium uptake than cells expressing the wild-type receptor [114]. Thus, the gain of function resulting from the H270R mutation suggests by inference that the human P2X7 receptor carrying His^27^^0^ or the R270H mutation is hypofunctional compared to the wild-type receptor carrying Arg^27^^0^. A recent study of the canine P2X7 receptor has shown the R270C mutant receptor was non-functional, highlighting the functional importance of Arg^27^^0^ [121]. The residue at position 270 is part of a loop region in the lower body of the extracellular domain which undergoes some conformational change during receptor activation (Figure 4E), but such information is insufficient to explain how the aforementioned mutations alter the receptor function.

### 3.8. A348T and the Human P2X7 Receptor

The A348T mutation was initially reported to decrease the functional activity of the human P2X7 receptor endogenously expressed in lymphocytes and heterologously expressed in HEK293 cells [117]. Subsequent studies have proved the contrary. Expression of the A348T mutant receptor in HEK293 cells resulted in significantly greater ATP-induced currents or dye uptake than when the wild-type human P2X7 receptor was expressed [113,119]. The mutation did not, however, have an effect on the ATP sensitivity. The equivalent residue in the rat P2X7 receptor is threonine and the introduction of the T348A mutation significantly reduced ATP-induced currents without altering the ATP sensitivity [119]. Immunofluorescent confocal imaging and Western blotting analyses indicate that the A348T mutation in the human P2X7 receptor and the reciprocal T384A mutation in the rat P2X7 receptor had no effect on the total and cell surface expression of the receptors [119], although a recent study suggests that the A348T mutation may also increase total protein expression of the human P2X7 receptor [120]. Homology modelling of the P2X7 receptors has revealed that this residue is located in TM2 as part of the intracellular pore and immediately adjacent to the physical gate (Figure 4F). There is evidence suggesting that the intracellular pore may narrow during channel opening [122]. As such it is likely that Ala^348^ plays a role in modulating channel opening, ion permeation or both. This notion is highly consistent with the inverse relationship between the size of the amino acid residue introduced at this position and the effect on the ATP-induced current amplitude; changes to residues with smaller side-chains resulted in larger currents, whereas changes to residues with much larger side-chains led to smaller currents [119].

### 3.9. Q460R and the Human P2X7 Receptor

The glutamine residue at position 460 is located in the intracellular *C*-terminus and is highly conserved across the mammalian P2X7 receptors. The ATP-induced currents in HEK293 cells expressing the Q460R mutant receptor were not statistically different from those in cells expressing the wild-type receptor [113,117]. Studies have reported that the Q460R mutation either had no effect [113,117], a slight increase [115] or a modest but significant reduction [114] in terms of the ability of the receptor to induce large pore formation or dye uptake. The findings that the Q460R mutation does not significantly affect P2X7 receptor function are perhaps rather unexpected, given the association of the 1405A>G polymorphism for this mutation with several disease conditions (Table 1). However, this NS-SNP has been found to be co-inherited with other NS-SNPs that confer gain-of-function mutations, such as H155Y, H270R, and A468T [114]. Further studies are thus required to examine the possibility that the disease association is related to the NS-SNPs that are co-inherited with the 1405A>G polymorphism.

### 3.10. E496A and the Human P2X7 Receptor

The E496A mutation resulting from the 1315A>C polymorphism is located in the intracellular *C*-terminal tail of the P2X7 receptor. This mutation has been examined in several studies and the literature contains conflicting results. An early immunofluorescent confocal microscopy study revealed similar cell surface expression for the wild-type receptor and the E496A mutant receptor endogenously expressed in human leukocytes [123]. However, further studies which made use of flow cytometry assays to determine ethidium dye uptake and Ba^2+^ influx in these cells showed that the mutant receptor was non-functional when expressed at low density but regained the normal functionality when expressed at high density [117,123]. HEK293 cells expressing the mutant receptor displayed very low ATP-dependent ethidium uptake [117], consistently supporting the idea that the large pore functionality of the P2X7 receptor is impaired. A separate study showed very similar ion channel functional properties as evidenced by the lack of significant difference in the ATP-induced current amplitude, channel activation and deactivation kinetics, and permeation between the wild-type and E496A mutant receptors expressed in *Xenopus* oocytes and HEK293 cells [124]. This study did not examine the large pore functionality but, by combining with the previous study [123], has concluded that Glu^496^ plays a determinant role in large pore formation but not in ion channel functional properties of the human P2X7 receptor. A subsequent study compared the ATP-induced currents and dye uptake mediated by the wild-type and E496A mutant receptors expressed in HEK293 cells, and the results clearly indicate that the E496A mutation impairs both ion channel and large pore functionalities [113].

The E496A mutation has been studied for its effect on the P2X7-dependent release of IL-1β. An earlier study showed that ATP-induced IL-1β release was attenuated in human monocytes expressing the E496A receptor upon treatment with a high concentration of ATP for short durations [125]. A recent study has used whole blood samples from patients with bone fractures carrying the E496A mutant receptor and from control subjects expressing the wild-type receptor, and found IL-1β release induced by prolonged exposure to ATP was in fact increased in cells expressing the E496A mutant receptor [126]. The exact cause for the discrepancy in such results remains to be established.

### 3.11. I568N and the Human P2X7 Receptor

The Ile^568^ residue lies in the distal tail of the intracellular *C*-terminus and is highly conserved among the mammalian P2X7 receptors. Human lymphocytes heterozygous for the I568N mutation showed lower ATP-induced Rb^+^ and Ba^2+^ flux (reflective of K^+^ and Ca^2+^ permeation respectively), due to reduced cell surface expression [127]. However, HEK293 cells expressing the P2X7 receptor containing this mutation exhibited no detectable ATP-evoked current and dye uptake [113], consistent with Ile^568^ being part of the trafficking motif that is composed of residues 551–581 and is important in membrane trafficking of the P2X7 receptor [128].

## 4. Concluding Remarks

Studies over the past decades, mainly using rodent animals, cells and models of diseases, have revealed an important role for P2X receptors in mediating extracellular ATP signalling in a wide range of physiological and pathological processes. The *P2RX* genes encoding the human P2X receptors are rich in SNPs. As highlighted above, an increasing number of NS-SNPs have shown to be associated with diseases and significantly influence the receptor activity by altering receptor expression and/or functional properties. These findings, taken together, have provided direct evidence to support a role of the human P2X receptors in health and disease. The evidence from these studies is compelling for certain diseases, but additional well-designed and large-scale genetic association studies are required to ascertain the role for the human P2X receptors in many other conditions. The study of NS-SNPs will continue to help us to advance the understanding of several aspects of the P2X receptors. Firstly, studies of SNPs in the *P2RX* genes can yield direct evidence for the role of human P2X receptors in the aetiology of a particular disease, such as the P2X2 receptor in hearing loss, or a group of diseases such as the P2X7 receptor in several inflammatory conditions. Secondly, disease-associated NS-SNPs could be used as diagnosis biomarkers to formulate preventive measurements, for example to reduce the risk of hearing loss and bone fracture and also to develop personalized treatments for osteoporosis and chronic pain. Thirdly, characterizations of the mutations caused by the disease-associated NS-SNP will aid us to gain a better understanding of the disease mechanisms and structure-function relationships of human P2X receptors. Finally, an improved delineation of the structure-function relationships of the human P2X receptors is critical for drug discovery because there are striking differences in functional and pharmacological properties between the human and rodent P2X receptors, as shown for the P2X2, P2X4, P2X5, and P2X7 receptors (e.g., [29,129,130,131,132,133,134,135]).
